# Lifestyle Behaviors and Suicide-Related Behaviors in Adolescents: Cross-Sectional Study Using the 2019 YRBS Data

**DOI:** 10.3389/fpubh.2021.766972

**Published:** 2021-11-19

**Authors:** Xiaozhi Li, Guijun Chi, Alyx Taylor, Si-Tong Chen, Aamir R. Memon, Yanjie Zhang, Yagang Song, Jinming Li, Xun Luo, Liye Zou

**Affiliations:** ^1^Department of Physical Education, Southeast University, Nanjing, China; ^2^China Volleyball College, Beijing Sport University, Beijing, China; ^3^Department of Physical Education, Tangshan Normal University, Tangshan, China; ^4^School of Rehabilitation, Sport and Psychology, AECC University College, Bournemouth, United Kingdom; ^5^Institute for Health and Sport, Victoria University, Melbourne, VIC, Australia; ^6^Institute of Physiotherapy and Rehabilitation Sciences, Peoples University of Medical and Health Sciences for Women, Nawabshah, Pakistan; ^7^Health and Exercise Science Laboratory, Institute of Sports Science, Seoul National University, Seoul, South Korea; ^8^Physical Education Unit, School of Humanities and Social Science, The Chinese University of Hong Kong-Shenzhen, Shenzhen, China; ^9^Department of Physical Education Teaching, Shanghai Sanda University, Shanghai, China; ^10^Exercise Psychophysiology Laboratory, Institute of KEEP Collaborative Innovation, School of Psychology, Shenzhen University, Shenzhen, China

**Keywords:** lifestyle, behaviors, suicide, adolescents, the Youth Risk Behavior Surveillance System Survey

## Abstract

**Objective:** The purpose of this research was to investigate the prevalence of lifestyle behaviors and suicide-related behaviors and the association between them using a nationally representative sample of adolescents from the USA.

**Methods:** 13,677 high school students aged 14-18 years were included in this cross-sectional study. The research data were retrieved from the Youth Risk Behavior Surveillance System Survey in 2019. All data on age, sex, grade, race, physical activity, television time, fruit intake, and suicide-related behavior were self-reported by students. Logistic regression models were adopted to examine the association between lifestyle behaviors and the suicide-related behaviors.

**Results:** Students who played video/computer games for ≥2 h had higher risk of suicide attempt (OR = 1.55, 95%CI: 1.30-1.85). Daily sleep duration of ≤8 h was positively associated with considering a suicide attempt (OR = 1.99, 95%CI: 1.62-2.43). In addition, participants who did not engage in any sport team were more likely to report considering a suicide attempt (OR = 1.50, 95%CI: 1.24-1.81).

**Conclusion:** This research suggests that some lifestyle behaviors (e.g., time for video or computer use, sleep duration, sports team participation, regular breakfast intake, and substance use) are associated with increased risk of suicidal behavior and ideation in high school students. To identify the specific effect of multiple lifestyle factors in influencing the risk of suicide-related behaviors in high school students, longitudinal studies are warranted in future.

## Introduction

The World Health Organization (WHO) data suggests that suicide has become one of the major global public health issues. Suicide has become the fourth leading cause of death among adolescents aged between 15 and 19 years (1). A study undertaken in the United States of America (USA) showed that there was an increase by 24% in the rate of suicide from 1999 to 2014 (2). The most recent WHO data from 2019 reveals that globally over 700,000 individuals commit suicide every year (1). The loss of life not only affects the families and communities but also places the burden of care for those left behind on the healthcare system. For example, a recent study by Shepard et al. (3) indicated that suicide-related costs (including suicide attempts) in the United States had reached approximately $94 billion (3). Furthermore, the estimated economic costs of youth suicide in Australia was about $AUD 500 million. To this end, effective suicide prevention strategy is urgently needed.

The physiological and psychological mechanisms that explain why individuals commit suicide still remain largely unknown (4). Recently, researchers have investigated the relationship of different modifiable risk factors, including physical activity (PA), sedentary behavior (e.g., video game or TV watching), adequate sleep duration, and sufficient nutrition with suicide-related behaviors (e.g., considering suicide, attempted suicide, and having a suicide plan). A recent meta-analytical review (5, 6) indicated an association between higher PA levels and lower suicidal ideation (7). Even low levels of PA was found to be associated with a lower risk for suicidal ideation (8). In contrast, sedentary adolescents have been found to have a greater risk for indulging in suicide-related behaviors (9, 10). In addition, other modifiable lifestyle behaviors including sleep duration, dietary habits (e.g., breakfast, fruit, and milk) (9, 11), and substance use (12, 13) have been studied in relation to suicide-related behaviors.

The above-mentioned lifestyle behaviors (e.g., PA, sedentary behavior, sleep duration, and/or dietary habits) have been shown to be associated with suicide-related behaviors, but the majority of previous studies have focused on one or two lifestyle behaviors or low-to-middle income countries, limiting researchers' comprehensive understanding of the associations between lifestyle behaviors and suicide-related behaviors. For example, a lifestyle study indicated that harmful consumption, sedentary behavior, and sexual risk behaviors were associated with the increased risk of suicide attempt among Namibia adolescents (14). It could be argued that multiple lifestyle behaviors act together and are likely to affect suicide-related behaviors. In addition, previous studies included multiple lifestyle behaviors, but outcomes of interest were depression, anxiety, and/or sleep quality (15, 16). Thus, investigation of associations between suicide-related behaviors and multiple lifestyle behaviors is required. To carry out timely and effective prevention strategies before suicide-related behaviors emerge, it is important to identify relevant multiple modifiable lifestyle factors associated with suicidal tendencies. Therefore, the aim of this study was to examine the prevalence of lifestyle behaviors and suicidal outcomes and the association between them in a nationally representative sample of adolescents from the USA.

## Methods

### Study Design and Participants

Data was retrieved from a project entitled the “Youth Risk Behavior Surveillance System (YRBS).” This biennially conducted project was designed by the Centre for Disease Control (CDC), USA, to track how risk behaviors change over time among high-school students. The YRBS survey was approved by the Institutional Review Board of the CDC. The sampling process consisted of three stages through which 9-12 grade students (14-18 years of age) from regular public school, parochial, and other non-public schools across the USA were approached for participation. The first-stage sampling frame comprised 1,257 primary sampling units (PSUs). The 1,257 PSUs were categorized into 16 strata. Among the 1,257 PSUs, 54 were sampled with probability proportional to overall school enrollment size for that PSU. For the second-stage sampling, secondary sampling units (SSUs) were defined as a physical school with grades 9–12 or a school created by combining nearby schools to provide all four grades. From the 54 PSUs, 162 SSUs were sampled with probability proportional to school enrollment size. To provide adequate coverage of students in small schools, an additional 15 small SSUs were selected from a subsample of 15 PSUs from the 54 PSU sample. These 177 SSUs corresponded to 184 physical schools. The third stage of sampling comprised random sampling of one or two classrooms in each of grades 9–12 from either a required subject (e.g., English or social studies) or a required period (e.g., homeroom or second period). All students in sampled classes were eligible to participate. Schools, classes, and students who refused to participate were not replaced in the sampling design. Data collected in 2019 included 13,872 participants from 136 schools invited to participate in the survey. Of these, 13,677 provided valid data for the final analysis. The overall response rate of the 2019 YRBS survey was 60.3%, calculated by multiplying school response rate (75.1%) with the student response rate (80.3%). Weighting for non-response and oversampling of minority students was carried out for each participant based on gender, ethnicity, and grade. A weight based on student sex, race/ethnicity, and grade was applied to each record to adjust for school and student non-response and oversampling of black and Hispanic students. The overall weights were scaled so that the weighted count of students equals the total sample size, and the weighted proportions of students in each grade match the national population proportions. Therefore, weighted estimates are nationally representative of all students in grades 9–12 attending U.S. public and private schools. The detailed sampling strategy is described in a previous study (17).

### YRBS Procedures

The YRBS survey was conducted at the targeted schools where informed consent was obtained and the legal guardian or parents signed the consent form. The participants were asked to respond to the computer-scannable questionnaire with assistance of trained data collectors. All procedures of the survey were designed based on the personal privacy protection, encouraging anonymous and voluntary participation, and all the study participants were informed about the research aims and given instructions prior to the formal survey.

### Measures

#### Independent Variables (Lifestyle Behaviors)

In the current study, physical activity (including muscle strengthening exercise, sport team participation), television viewing, video [or computer games] use, sleep duration, physical education, fruit intake, vegetable intake, milk consumption, eating breakfast, and substance use (including current alcohol use and smoking) were included as independent variables. The details of measures used to assess lifestyle behaviors are available in Supplementary Table 1.

#### Outcome Variables (Suicide-Related Behaviors)

Three suicide-related behaviors (i.e., considered attempting suicide, planned suicide, and attempted suicide) were included in the current study. Considered attempting suicide was asked through a question with dichotomous answer (*yes* or *no*): “During the past 12 months, did you ever seriously consider attempting suicide?”; planned suicide was confirmed through the question (with *yes* or *no* responses): “During the past 12 months, did you make a plan about how you would attempt suicide?”; and attempted suicide was confirmed from the question “During the past 12 months, how many times did you actually attempt suicide?” with responses ranging from 0 to 6 or more times. For attempted suicide, study participants answering 0 times were considered as *no* suicide attempt.

#### Control Variables (Demographic Factors)

Study participants reported their demographic characteristics, including sex (female or male), age (ranging from ≤12 to ≥18 years), grade (from 9th to 12th), race (white, African American, Hispanic/Latino or all other races). Likewise, participants self-reported their height (feet and inches) and weight (pounds) on a paper-based questionnaire, which were used to calculate body mass index so that overweight or obesity status could be determined. All these variables were considered as confounding variables during statistical analysis.

### Statistical Analysis

Following the YRBS protocol, all the statistical analysis were performed after taking the complex sampling design into account to estimate nationally representative results of included variables. Missing data were not imputed. The statistical analysis was performed using SPSS v26.0. All the variables were treated as categorical ones; thus, descriptive statistics (percentage, %) were calculated to report the characteristics of the study sample (Supplementary Table 1). The weighted percentage of each variable was also reported with 95% confidence interval. Binary logistic regression was utilized to assess associations of lifestyle behaviors with suicidal outcomes while controlling for sex, age, grade, race, overweight and obesity. In the models, those reporting *no* for the outcome variable were considered the reference group, whereas the reference group for each independent variable was as follows: sufficient physical activity (yes), television watching hours (≤2 h per day), played video or computer games or used a computer (≤2 h per day), sleep duration was >8 h per night (yes), physical education attendance for at least 1 day (yes), muscle strengthening exercise for at least three times a week (yes), participation in team sports for at least one team (yes), do not eat fruit (no), not eat vegetable (no), do not eat breakfast (no), do not drink milk (no), currently use alcohol (no), and currently smoking (no). The odds ratios with 95%CI were calculated to assess the associations of lifestyle behaviors with the suicidal outcomes. The above statistical analysis was performed using the Complex Sample Module of the SPSS. Statistical significance was set as *p* < 0.05.

## Results

Participant characteristics are shown in Table 1. Of the 13,677 participants, 50.3% were female. The age range 15-17 years made up the majority of the participants (74.6%). The proportions of 9th, 10th, 11th, and 12th grade students were 26.6, 27.2, 24.3, and 20.8%, respectively. The proportion of White was highest across all the race groups (48.8%). The prevalence of overweight (14.4%) and obesity (13.1%) were relatively equal.

**Table 1:** Demographic characteristics of the participants.

		***n***	**%**	**Weighted %**	**95%CI**	
Total		13,677	100	/	/	/
**Sex**						
	Female	6,885	50.3	49.4	47.9	50.9
	Male	6,641	48.6	50.6	49.1	52.1
	Missing	151	1.1			
**Age group**						
	≤12 years	60	0.4	0.3	0.2	0.5
	13 years	27	0.2	0.1	0.0	0.2
	14 years	1,699	12.4	11.9	10.9	13.0
	15 years	3,473	25.4	24.8	23.5	26.0
	16 years	3,628	26.5	25.6	24.5	26.7
	17 years	3,102	22.7	23.7	22.5	24.8
	≥18 years	1,616	11.8	13.7	12.6	14.9
	Missing	72	0.5			
**Grade**						
	9th	3,637	26.6	26.6	25.4	28.0
	10th	3,717	27.2	25.5	24.7	26.3
	11th	3,322	24.3	24.3	23.2	25.4
	12th	2,850	20.8	23.6	22.4	24.8
	Missing	151.0	1.1			
**Race**						
	White	6,668	48.8	51.2	46.4	56.0
	African American	2,040	14.9	12.2	10.2	14.6
	Hispanic/Latino	3,038	22.2	26.1	21.8	30.9
	All other races	1,493	10.9	10.5	7.9	13.9
	Missing	438	3.2			
**Overweight**						
	Yes	1,933	14.1	16.10	14.90	17.50
	No	10,207	74.6	83.90	82.50	85.10
	Missing	1,537	11.2			
**Obesity**						
	Yes	1,795	13.1	15.50	13.80	17.30
	No	10,345	75.6	84.50	82.70	86.20
	Missing	1,537	11.2			

*CI, confidence interval*.

Weighted results of lifestyle behaviors are presented in Table 2. Only 21.6% of the participants reached the sufficient level of physical activity. Television viewing and use of video/computer games for <2 h per day accounted for 74.6 and 53.0% of the participants, respectively. Only 21.4 % of the participants slept for >8 h per night. Attendance of physical education for at least 1 day was reported by 39.7% participants. Moreover, 30.1% of the participants engaged for >3 days in muscle strengthening exercise. Over two-fifths of the participants participated in at least one sport team. The prevalence of participants who did not eat/drink fruit, vegetable, breakfast and milk were 11.9, 7.3, 14.3, and 21.3%, respectively. Current alcohol use was reported by 26.8% of participants and current smoking by 5.3%.

**Table 2:** Prevalence of lifestyle behaviors and suicide-related behaviors.

		***N***	**%**	**Weighted %**	**95%CI**	
Total		13,677	100	/	/	/
**Sufficient physical activity**						
	Yes	2,954	21.6	23.2	21.9	24.6
	No	10,266	75.1	76.8	75.4	78.1
	Missing	457	3.3			
**Television watching hours**						
	Yes	10,200	74.6	80.2	78.7	81.7
	No	2,596	19.0	19.8	18.3	21.3
	Missing	881	6.4			
**Played video or computer games or used a computer**						
	Yes	7,246	53.0	53.9	52.1	55.6
	No	5,931	43.4	46.1	44.4	47.9
	Missing	500	3.7			
**Sleep for >8 h per night**						
	Yes	2,930	21.4	22.1	20.6	23.7
	No	10,175	74.4	77.9	76.3	79.4
	Missing	572	4.2			
**Physical education attendance (at least 1 days)**						
	Yes	5,423	39.7	52.2	46.9	57.4
	No	5,865	42.9	47.8	42.6	53.1
	Missing	2,389	17.5			
**Muscle strengthening exercise (at least three times a week)**						
	Yes	4,121	30.1	49.5	47.6	51.3
	No	4,353	31.8	50.5	48.7	52.4
	Missing	5,203	38.0			
**Participation in team sports (at least one team)**						
	Yes	5,545	40.5	57.4	54.3	60.4
	No	4,242	31.0	42.6	39.6	45.7
	Missing	3,890	28.4			
**Do not eat fruit**						
	Yes	1,631	11.9	11.9	10.5	13.4
	No	11,255	82.3	88.1	86.6	89.5
	Missing	791	5.8			
**Do not eat vegetable**						
	Yes	992	7.3	7.9	7.1	8.7
	No	10,765	78.7	92.1	91.3	92.9
	Missing	1,920	14.0			
**Do not eat breakfast**						
	Yes	1,956	14.3	16.7	15.3	18.1
	No	9,637	70.5	83.3	81.9	84.7
	Missing	2,084	15.2			
**Do not drink milk**						
	Yes	2,919	21.3	30.6	29.0	32.3
	No	6,570	48.0	69.4	67.7	71.0
	Missing	4,188	30.6			
**Currently alcohol use**						
	Yes	3,669	26.8	29.2	27.3	31.2
	No	8,942	65.4	70.8	68.8	72.7
	Missing	1,066	7.8			
**Currently smoking**						
	Yes	726	5.3	6.0	5.0	7.2
	No	11,591	84.7	94.0	92.8	95.0
	Missing	1,360	9.9			
**Consider attempting suicide**						
	Yes	2,633	19.3	18.8	17.6	20.0
	No	10,804	79.0	81.2	80.0	82.4
	Missing	240	1.8			
Make a suicide plan	Yes	2,151	15.7	15.7	14.6	16.9
	No	11,271	82.4	84.3	83.1	85.4
	Missing	255	1.9			
**Attempt suicide**						
	Yes	1,067	7.8	8.9	7.9	10.0
	No	9,453	69.1	91.1	90.0	92.1
	Missing	3,157	23.1			

*CI, confidence interval*.

Table 3 presents information on the associations of lifestyles behaviors with each outcome: consider attempting suicide, make a suicide plan and attempt suicide. Significant results were only observed in terms of video/computer games, sleep duration, sport team participation, eating/drinking behaviors (breakfast, alcohol use, and smoking). Specifically, participants who played video/computer games for ≥2 h were more likely to report considering a suicide attempt (OR = 1.55, 95%CI: 1.30-1.85). Daily sleep duration of ≤8 h was positively associated with considering a suicide attempt (OR = 1.99, 95%CI: 1.62-2.43). In addition, participants who did not engage in any sport team were more likely to report considering a suicide attempt (OR = 1.50, 95%CI: 1.24-1.81). Further, participants with an eating habit of no breakfast, current alcohol use and smoking habit had higher odds for considering a suicide attempt. Similar associations were found for other outcomes (i.e., suicidal plan and attempt suicide).

**Table 3:** Factors associated with suicide-related behaviors (consider attempting suicide, make a suicide plan and attempt suicide).

	**Consider attempting suicide (Yes)**			**Make a suicide plan (Yes)**			**Attempt suicide (Yes)**		
	**OR**	**95%CI**		**OR**	**95%CI**		**OR**	**95%CI**	
Sufficient physical activity (REF = yes)	1.18	0.97	1.44	1.07	0.82	1.40	1.08	0.79	1.48
Television watching hours (REF = no more than 2 h per day)	1.00	0.82	1.21	0.93	0.75	1.16	1.06	0.84	1.34
Played video or computer games or used a computer (REF = no more than 2 h per day)	**1.55**	**1.30**	**1.85**	**1.50**	**1.24**	**1.81**	1.23	0.91	1.68
Sleep more than 8 hours nightly (REF = yes)	**1.99**	**1.62**	**2.43**	**1.90**	**1.48**	**2.45**	**1.86**	**1.31**	**2.63**
Physical education attendance (at least 1 days) (REF = yes)	1.04	0.85	1.26	1.15	0.90	1.46	1.14	0.91	1.43
Muscle strengthening exercise (at least three times a week) (REF = yes)	1.07	0.90	1.28	0.87	0.71	1.06	0.74	0.55	1.01
Sport team participation (at least one team) (REF = yes)	**1.50**	**1.24**	**1.81**	**1.31**	**1.13**	**1.53**	**1.49**	**1.19**	**1.87**
Do not eat fruit (REF = no)	1.29	0.85	1.95	1.24	0.79	1.95	0.79	0.53	1.19
Do not eat vegetable (REF = no)	0.69	0.47	1.01	0.86	0.56	1.31	1.00	0.64	1.56
Do not eat breakfast (REF = no)	**1.39**	**1.14**	**1.71**	**1.49**	**1.12**	**1.98**	**1.64**	**1.18**	**2.27**
Do not drink milk (REF = no)	0.93	0.74	1.15	0.92	0.75	1.14	1.12	0.87	1.45
Currently alcohol use (REF = no)	**1.74**	**1.38**	**2.19**	**1.60**	**1.26**	**2.02**	**1.87**	**1.36**	**2.57**
Currently smoking (REF = no)	**2.20**	**1.70**	**2.85**	**2.17**	**1.53**	**3.08**	**2.86**	**1.94**	**4.21**

*REF, reference group; All models were controlled for sex, age, grade, race, overweight, and obesity; Bold numbers: statistically significant at p < 0.05*.

## Discussion

The current study was designed to investigate the prevalence and lifestyle correlates of suicide-related behaviors, which include suicidal consideration, suicidal plan, and suicidal attempt, using a nationally representative sample of US adolescents. The findings of the current study show that spending time on video or computer games (>2 h per day), insufficient nocturnal sleep (<8 h per day), and sport team participation (<1 team) were positively associated with higher risks of three suicide-related behaviors in adolescents, while sufficient PA was not correlated with suicide-related behaviors. Moreover, no eating/drinking behaviors but eating breakfast was negatively associated with suicide-related behaviors. Excessive substance use (alcohol and smoking use) were risk factors of suicide-related behaviors in adolescents.

To investigate the potential factors that influence suicide-related behaviors, most previous studies have examined only a few lifestyle behaviors (e.g., physical activity, sleep) (8, 18), and have not yet examined others. As such, previous research may offer limited results. In fact, suicide-related behavior is influenced by a multiplicity of risk factors (19), such as physical inactivity, poor sleep (20), sedentary behavior (7), substance abuse (21), and poor diet (11). It is, thus, essential to take these lifestyle factors together to investigate their relationship with suicide-related behaviors. Therefore, our study included additional factors such as participation in team sport, physical education attendance, and muscle strengthening exercise in relation to the suicide-related behaviors. Interestingly, the findings of our study showed that there were no significant associations of physical activity, television watching hours, physical education attendance, muscle strengthening exercise, and diet (e.g., eat fruit, eat vegetables, and drink milk) with suicide-related behavior. One plausible reason for this may be a combination of including other lifestyle behaviors and controlling for personal factors/potential confounders, which in turn attenuates the associations of physical activity, television watching hours, physical education attendance, muscle strengthening exercise and diet behaviors with suicide-related behaviors.

With respect to sleep, our finding was consistent with a previous study (22) which found that insufficient sleep was associated with a higher risk of suicide-related behaviors (e.g., consider suicide, and attempt suicide) among adolescents. One recent cross-sectional study showed a higher risk of suicide-related behaviors in those with insufficient sleep than those without sleep problems (21). It is possible that insufficient sleep may cause a decrease in the frontal lobe function and diminish the executive functions, which form a common pathway to the suicidal tendency in adolescents (23). Besides, insufficient sleep duration may result from poor sleep quality, which in turn is associated with suicide-related behaviors (24). Although sleep interventions have been proposed as potential prevention of suicide risk (25), the underlying mechanisms linking insufficient sleep and suicide-related behaviors among adolescents are still unclear, especially the underlying physiological pathways. Of note, the study could not determine the direction of the association between insufficient sleep and suicide outcomes. An alternative explanation for the significant association found in this study may be that those with suicidal ideas have been affected by negative emotions, which might have disturbed the sleep (26) and lead to insufficient sleep.

It is very common for modern teenagers to use electronic devices. Most students use electronic devices to access the internet and play games. The negative effects caused by improper use of electronic products have attracted many researchers' interest, who have found that playing games can result in psychological problems, which eventually leads to suicide-related behaviors among adolescents (27, 28). For instance, Rostad et al. found that excessive media use is associated with an increased risk of suicide-related behaviors among high school students (29). Overall, our findings have added to existing knowledge, indicating an association between the screen-based risk factors (watching videos, computer games, or computer use) and suicide-related behaviors. As the direction could not be determined in this study, prospective studies are needed to examine the relationships between screen-based behaviors and suicide-related behaviors.

The protective effects of sport participation on suicide-related behaviors have been indicated in the previous literature. Participation in team sports (at least one team) was associated with a lower risk of suicide-related behaviors in the present study, as observed in previous studies on adolescents (30, 31). The mechanism by which participation in sports reduces the risk of the three suicide-related behaviors is not yet clear. However, it could be argued that adolescents who engage in the sport can have chances to establish positive social relationships with participants of the activity (32). Adolescents with strong social support generally show higher resilience, less hopelessness, and a lower risk of suicide (30). As team sports participation has been one of the relatively prevalent forms of PA among adolescents in the USA, encouraging their participation in team sports is a feasible approach to reduce suicide-related behaviors. However, as this data was based on adolescents from the USA, future research should examine the associations between team sports participation and suicide-related behaviors in adolescents from other countries. Interestingly, the current study found that no association between PA and suicide-related behaviors in adolescents, which is inconsistent with previous studies using data from prior YRBS surveys (9, 33). A possible reason for this finding is that several lifestyle behaviors examined in the current study offset the independent effect of PA on suicide-related behaviors in adolescents.

Compared with eating breakfast, not eating breakfast was positively associated with a higher risk of suicide-related behaviors in adolescents. This finding indicates that eating breakfast might play a role in reducing the risks of suicide-related behaviors and ideation in adolescents. Although the existing literature has fewer studies on the role of breakfast on suicide-related behaviors, the association between not eating breakfast and higher risks for suicide-related behaviors and ideation can still be supported by the limited evidence (9, 34). A possible mechanism linking breakfast consumption with suicide-related behaviors is the positive effect of breakfast consumption on mood. Not eating breakfast is associated with poorer emotions, such as depression (35, 36) or anxiety (36–38), both of which are contributing factors for suicide-related behaviors and ideation in adolescents. Nevertheless, owing to the cross-sectional nature of this study, it is also possible that those with suicidal ideations or attempts may already have been affected by their negative emotions or other life difficulties, so they had low appetite and did not want to eat breakfast.

Current alcohol use and current smoking were two positive correlates of suicide-related behaviors in adolescents in this study. Thus, restricting access for adolescents to alcohol and cigarettes is recommended. Previous studies have suggested that alcohol use and smoking are associated with higher risks for suicide-related behaviors (39). Our research findings were that adolescents with current alcohol use had a greater likelihood of suicidal attempt, plans, and ideation, which agrees with previous evidence. It is well-established that excessive alcohol use is associated with dysfunction of the neuroendocrine system, which subsequently increases the risk for suicide-related behaviors in the general population (39). Concerning the association between smoking and suicide-related behaviors, a few previous studies reported findings similar to the current study. For example, a study of adolescents in Nepal found that smoking was associated with suicidal attempts (40). Similarly, cross-sectional evidence indicated that more smoking was associated with suicidal ideation (41), a suicidal plan (42), and suicidal attempts among Korean adolescents (43). In a meta-analysis, the authors found that smoking is associated with an increased risk of suicide-related behaviors and ideation (44). There are plausible explanations for the associations between smoking and suicide-related behaviors. One is that smokers have pre-existing conditions that result in higher risks for suicide, alternatively that smoking causes adverse conditions that probably lead to the development of suicidal tendency (45). However, these explanations remain untested. It could also be argued that individuals with suicide-related behaviors (e.g., suicide ideation) may be more likely to engage in drinking or smoking behaviors. While the direction of the association between cigarette smoking and suicide-related behaviors remains unclear, it has been argued that the co-occurance deserves attention (46). From the perspective of public health, the possibility that the use of alcohol and / or cigarettes may contribute to suicide-related behaviors suggests that policies reducing adolescents' exposure to alcohol and cigarette products are needed.

### Study Strengths and Limitations

The current study has two strengths. The first one is that we used a nationally representative sample of adolescents, which can increase the generalizability of research findings. Second, we included several lifestyle factors to assess the associations between lifestyle behaviors and suicidal outcomes, after controlling for multiple demographic factors, in order to determine the specific role of each behavior in affecting suicidal tendencies. However, some study limitations should be mentioned. One weakness is that the YRBS survey has a cross-sectional study design, which prevents the possibility of establishing a causal association. Moreover, the use of self-reported measures can be subject to reporting and recall bias due to social desirability. Furthermore, owing to the standardized measurement protocol, each variable in our study was assessed by a single item and the measures may be negatively impacted owing to limited validity and reliability.

### Practical Implications

The current study has practical implications guiding suicide-related interventions. Our results indicate the potential importance of lifestyle behaviors for prevention of suicide. This calls for the need to raise public health awareness on promoting healthy lifestyle behaviors of adolescents. The results of our study may also specifically stress the role of changing lifestyle behaviors in preventing suicide. For example, discouraging substance use (including alcoholism and smoking) may be an effective approach to reduce suicide ideation and behavior. Similarly, from the perspective of changing movement behaviors, reducing time for games (video or computer) and encouraging activity through team sports are two potential ways to decrease the risk of suicide-related behaviors. Collectively, considering that the current study did not use a causal inference approach to identify the aforementioned associations, addressing suicide-related behaviors through improving their lifestyle is a potential way that needs more examination.

## Conclusion

This study suggests that optimal lifestyle behaviors may have an essential role in preventing suicidal behavior and ideation in adolescents. For example, limiting time for video or computer use, encouraging adequate sleep duration, sport team participation, regular breakfast consumption, and avoiding substance use might be protective factors against suicide-related behaviors. Owing to the study design, longitudinal studies are needed in the future to determine the specific effect of multiple lifestyle factors on reducing the risk of suicide-related behaviors in adolescents.

## Data Availability Statement

Publicly available datasets were analyzed in this study. This data can be found at: The data presented in this study are available on request from https://www.cdc.gov/healthyyouth/data/yrbs/index.htm (accessed on July, 23 2021).

## Ethics Statement

YRBS survey was approved by the Institutional Review Board of the Centre for Disease Control (CDC), USA. Written informed consent to participate in this study was provided by the participants' legal guardian/next of kin.

## Author Contributions

XL and GC: conceptualization. XL: methodology, formal analysis, investigation, and writing—original draft preparation. GC and S-TC: validation. YS: resources, supervision, and project administration. All authors: writing—review and editing.

## Conflict of Interest

The authors declare that the research was conducted in the absence of any commercial or financial relationships that could be construed as a potential conflict of interest.

## Publisher's Note

All claims expressed in this article are solely those of the authors and do not necessarily represent those of their affiliated organizations, or those of the publisher, the editors and the reviewers. Any product that may be evaluated in this article, or claim that may be made by its manufacturer, is not guaranteed or endorsed by the publisher.
